# Mex‐3 RNA‐binding family member A limits macrophage ferroptosis‐associated injury linked to the SLC7A11/GPX4 pathway in diabetic atherosclerosis

**DOI:** 10.1002/ctm2.70725

**Published:** 2026-06-22

**Authors:** Yanpeng Ma, Jing Liu, Yujie Xing, Shuo Pan, Begench H. Annayev, We Wu, Xiqiang Wang, Zhongwei Liu

**Affiliations:** ^1^ Department of Cardiology Shaanxi Provincial People's Hospital Xi'an China; ^2^ Shaanxi Cardiovascular and Cerebrovascular Disease Precision Medicine Belt and Road Joint Laboratory Xi'an China; ^3^ Hospital with Scientific Clinical Centre of Cardiology Ashgabat Turkmenistan; ^4^ School of Life Sciences and Technology Northwestern Polytechnical University Xi'an China

**Keywords:** diabetic atherosclerosis, ferroptosis, GPX4, macrophage, MEX3A, plaque stability, SLC7A11

## Abstract

**Background:**

Diabetes accelerates atherosclerotic plaque expansion and loss of stability, but the plaque‐resident mechanisms through which the diabetic milieu promotes macrophage lipid‐oxidative injury are not fully understood.

**Methods:**

We used apolipoprotein E‐deficient (*ApoE* ^−/−^) mice with Mex‐3 RNA‐binding family member A (MEX3A) deficiency and primary bone marrow‐derived macrophage (BMDM) experiments under diabetic conditions to investigate the role of MEX3A in diabetic atherosclerosis and macrophage lipid‐peroxidation injury.

**Results:**

MEX3A loss worsened diabetic atherosclerosis while leaving body weight, glycaemia and lipid measurements largely unchanged relative to diabetes alone. MEX3A deficiency enlarged aortic lesions, enhanced lipid deposition, increased macrophage content, expanded necrotic cores and thinned fibrous caps, while reducing collagen content. In lesional macrophages, loss of MEX3A coincided with increased lipid reactive oxygen species, malondialdehyde, increased labile iron, 4‐hydroxynonenal, a disturbed glutathione redox state, mitochondrial abnormalities compatible with ferroptotic stress, lower GPX4/SLC7A11 and higher ACSL4. CRISPR/Cas9‐mediated *Mex3a* knockout in BMDMs recapitulated this phenotype, whereas restoring MEX3A blunted it. RNA immunoprecipitation‐qPCR together with actinomycin D decay analyses demonstrated preferential recovery of *Slc7a11* and *Gpx4* transcripts with MEX3A and decreased stability of *Slc7a11* and *Gpx4* mRNAs after *Mex3a* knockout. GPX4 or SLC7A11 overexpression reduced lipid‐peroxidation injury in *Mex3a*‐knockout macrophages, whereas *Gpx4* or *Slc7a11* knockdown weakened the protection conferred by MEX3A re‐expression. Ferrostatin‐1 partially attenuated macrophage lipid peroxidation and plaque injury.

**Conclusions:**

Together, these results place MEX3A among the protective regulators of diabetic plaque stability and support a MEX3ASLC7A11/GPX4‐linked ferroptosis‐associated mechanism in plaque macrophages.

**Key points:**

- MEX3A deficiency worsens diabetic atherosclerosis without further aggravating systemic metabolic indices.
- Loss of MEX3A promotes plaque lipid deposition, macrophage accumulation, necrotic core expansion and fibrous cap thinning.
- MEX3A limits macrophage lipid‐peroxidation injury linked to the SLC7A11/GPX4 pathway. Ferrostatin‐1 partially attenuates macrophage lipid peroxidation and plaque injury associated with MEX3A deficiency.

## Graphical abstract

1. Diabetes reduces macrophage MEX3A and aggravates atherosclerotic plaque instability.
2. MEX3A stabilises Slc7a11 and Gpx4 mRNAs to sustain SLC7A11/GPX4 antioxidant defence.
3. MEX3A deficiency promotes macrophage ferroptosis, lipid peroxidation and mitochondrial injury.
4. Ferrostatin‐1 partially attenuates ferroptotic injury and diabetic plaque destabilisation.

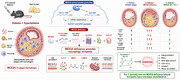

## 1 INTRODUCTION

Atherosclerosis in diabetes is marked by faster lesion expansion together with plaques that contain more inflammatory cells and lipid and are more prone to rupture.^1^, ^2^ Hyperglycaemia, dyslipidaemia and redox stress reshape the arterial niche and heighten immune activity in the vessel wall.^2^ These changes weaken the structural elements that maintain plaque integrity and promote lesion progression.^3^ Yet the plaque‐intrinsic determinants that translate diabetic stress into destabilising cellular programmes remain insufficiently resolved.

Macrophages sit at the centre of this process. They control lipid uptake, inflammatory escalation, necrotic‐core expansion and remodelling of the fibrous cap across the full life cycle of the plaque.^4^ The phenotype and fate of lesional macrophages are therefore key determinants of whether plaques remain stable or acquire vulnerable features.^5^ In addition to apoptosis, regulated necrotic pathways, including ferroptosis, are increasingly recognised as contributors to plaque progression because they couple oxidative injury to inflammatory tissue damage.^6^, ^7^

Ferroptosis denotes a regulated cell‐death pathway driven by iron‐dependent lipid peroxide accumulation characterised by failed detoxification of phospholipid peroxides and distinctive mitochondrial injury, including shrinkage and cristae loss.^8^, ^9^ SLC7A11, a component of system Xc‐, supports glutathione production, whereas GPX4 removes phospholipid hydroperoxides.^10^, ^11^ ACSL4 promotes incorporation of polyunsaturated phospholipids into membranes, thereby increasing ferroptosis susceptibility.^12^ Increasing evidence connects ferroptosis with cardiovascular pathology, including atherosclerotic lesions.^13^, ^17^ However, the upstream regulators that determine macrophage ferroptosis‐associated injury in diabetic plaques remain incompletely mapped.

Mex‐3 RNA‐binding family member A (MEX3A) is a conserved RNA‐binding protein that contains two K‐homology domains and a carboxy‐terminal RING finger, features that link it to post‐transcriptional regulation and ubiquitin‐associated processes.^14^ Although MEX3A has been investigated primarily in stem‐cell and cancer contexts,^15^ recent evidence indicates an atheroprotective role in vivo and genetic association with human coronary artery disease.^16^ Because RNA‐binding proteins can coordinate stress‐responsive transcript stability, translation and inflammatory cell fate, MEX3A is a plausible regulator of macrophage responses to diabetic oxidative stress. Direct evidence connecting MEX3A with macrophage ferroptosis remains scarce, so we therefore investigated whether MEX3A regulates ferroptosis‐associated macrophage injury during diabetic plaque progression.

Here, we show that MEX3A deficiency aggravates diabetic atherosclerosis, enhances macrophage lipid‐peroxidation injury and destabilises plaques. We further demonstrate that CRISPR/Cas9‐mediated *Mex3a* knockout and MEX3A re‐expression in bone marrow‐derived macrophages (BMDMs) identify a *Mex3a*‐dependent macrophage phenotype, that MEX3A associates with *Slc7a11* and *Gpx4* transcripts and supports their mRNA stability, and that GPX4/SLC7A11 function as downstream mediators of the MEX3A‐associated phenotype. Finally, Ferrostatin‐1 (Fer‐1) partially attenuates, but does not specifically rescue, the MEX3A‐deficient phenotype, indicating that ferroptosis‐associated injury contributes to the pathology while additional pathways may also be involved.

## 2 RESULTS

### 2.1 MEX3A deficiency aggravates diabetic atherosclerosis without further worsening systemic metabolic indices

**FIGURE 1:**
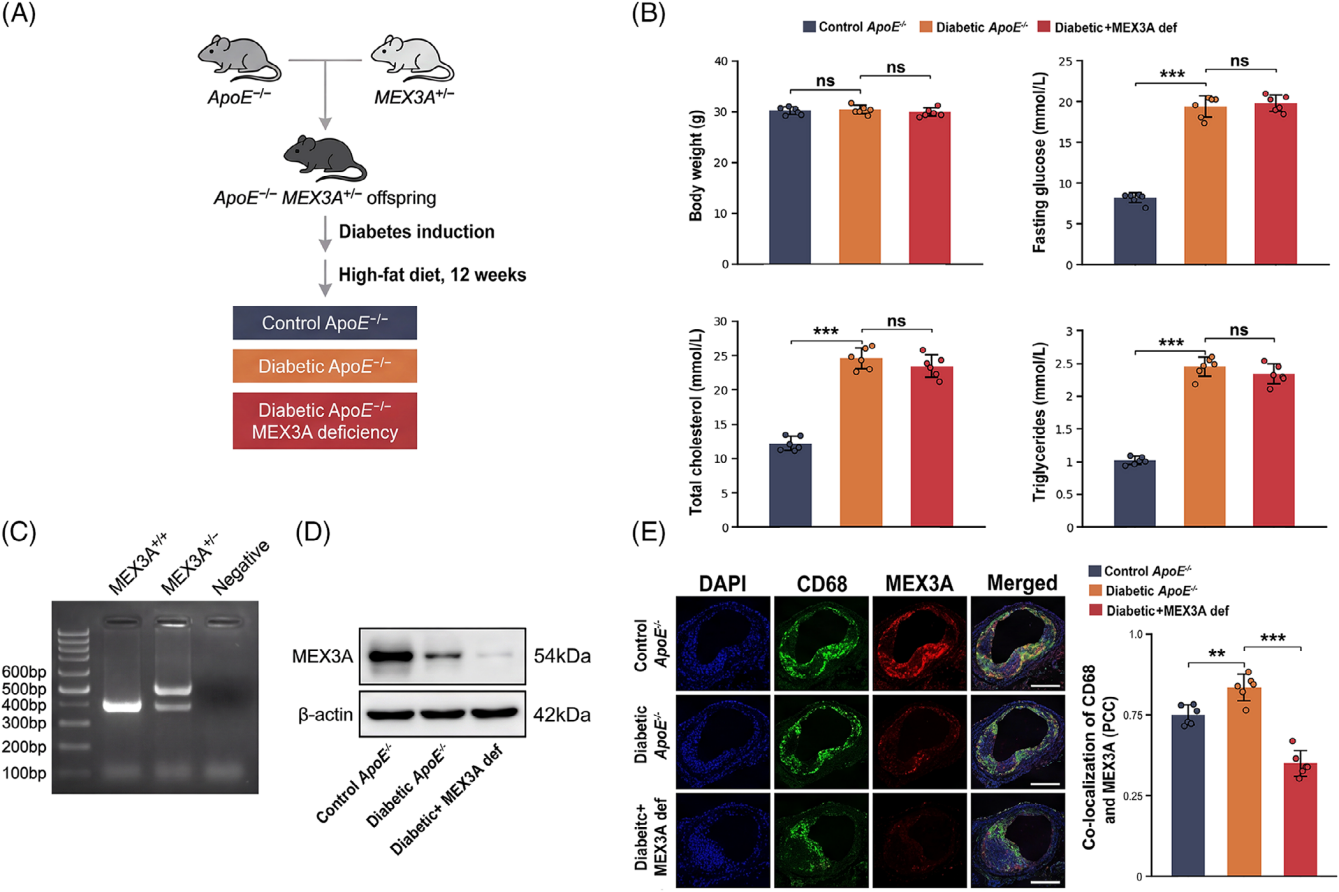
Generation of the diabetic atherosclerosis model with Mex‐3 RNA‐binding family member A (MEX3A) deficiency and baseline characterisation. (A) Schematic of the in vivo experimental design. *ApoE* ^−/−^ mice were crossed with *Mex3a* ^+/−^ mice to generate *ApoE* ^−/−^ *Mex3a* ^+/−^ offspring. Diabetes was induced with streptozotocin (STZ), followed by high‐fat diet (HFD) feeding for 12 weeks. (B) Body weight, fasting glucose, total cholesterol and triglycerides in control *ApoE* ^−/−^, diabetic *ApoE* ^−/−^ and diabetic + MEX3A def mice. *n* = 6 mice per group. (C) PCR genotyping of *Mex3a* alleles. (D) Western blot analysis of MEX3A protein in aortic tissues; beta‐actin was used as loading control. (E) Representative immunofluorescence images showing 4′,6‐diamidino‐2‐phenylindole (DAPI) (blue), CD68 (green), MEX3A (red) and merged signal in aortic root plaques. Right, quantification of CD68/MEX3A co‐localisation by Pearson correlation coefficient (PCC). Scale bars, 200 µm. Data are mean ± standard deviation (SD). One‐way analysis of variance (ANOVA) with Tukey's post hoc test. ns, not significant; ^**^ *p* < .01; ^***^ *p* < .001.

To define the contribution of MEX3A to diabetic atherogenesis, *ApoE* ^−/−^ mice were crossed with *Mex3a* heterozygous knockout mice to generate *ApoE* ^−/−^ *Mex3a* ^+/−^ offspring and were subjected to streptozotocin (STZ)‐induced diabetes followed by a 12‐week high‐fat diet (HFD) challenge (Figure 1A). Male mice were used. The design enabled comparison among non‐diabetic *ApoE* ^−/−^ controls, diabetic *ApoE* ^−/−^ mice and diabetic *ApoE* ^−/−^ mice with MEX3A deficiency. Body weight did not differ significantly among groups, whereas fasting glucose, total cholesterol and triglycerides were markedly increased by diabetes. Importantly, MEX3A deficiency did not further increase fasting glucose, total cholesterol or triglycerides relative to diabetic *ApoE* ^−/−^ controls (Figure 1B). PCR genotyping verified the expected alleles, and immunoblotting confirmed reduced MEX3A protein abundance in aortic tissue from MEX3A‐deficient mice (Figure 1C,D). Immunofluorescence further showed that MEX3A co‐localised with CD68‐positive plaque macrophages and that macrophage‐associated MEX3A was diminished by diabetes and more prominently by genetic MEX3A deficiency (Figure 1E). These findings indicate that the intensified vascular phenotype observed after MEX3A deficiency is unlikely to be explained by more severe systemic metabolic derangement and support plaque macrophages as a relevant site of MEX3A action.

### 2.2 MEX3A deficiency increases plaque burden and promotes morphological features of plaque vulnerability

**FIGURE 2:**
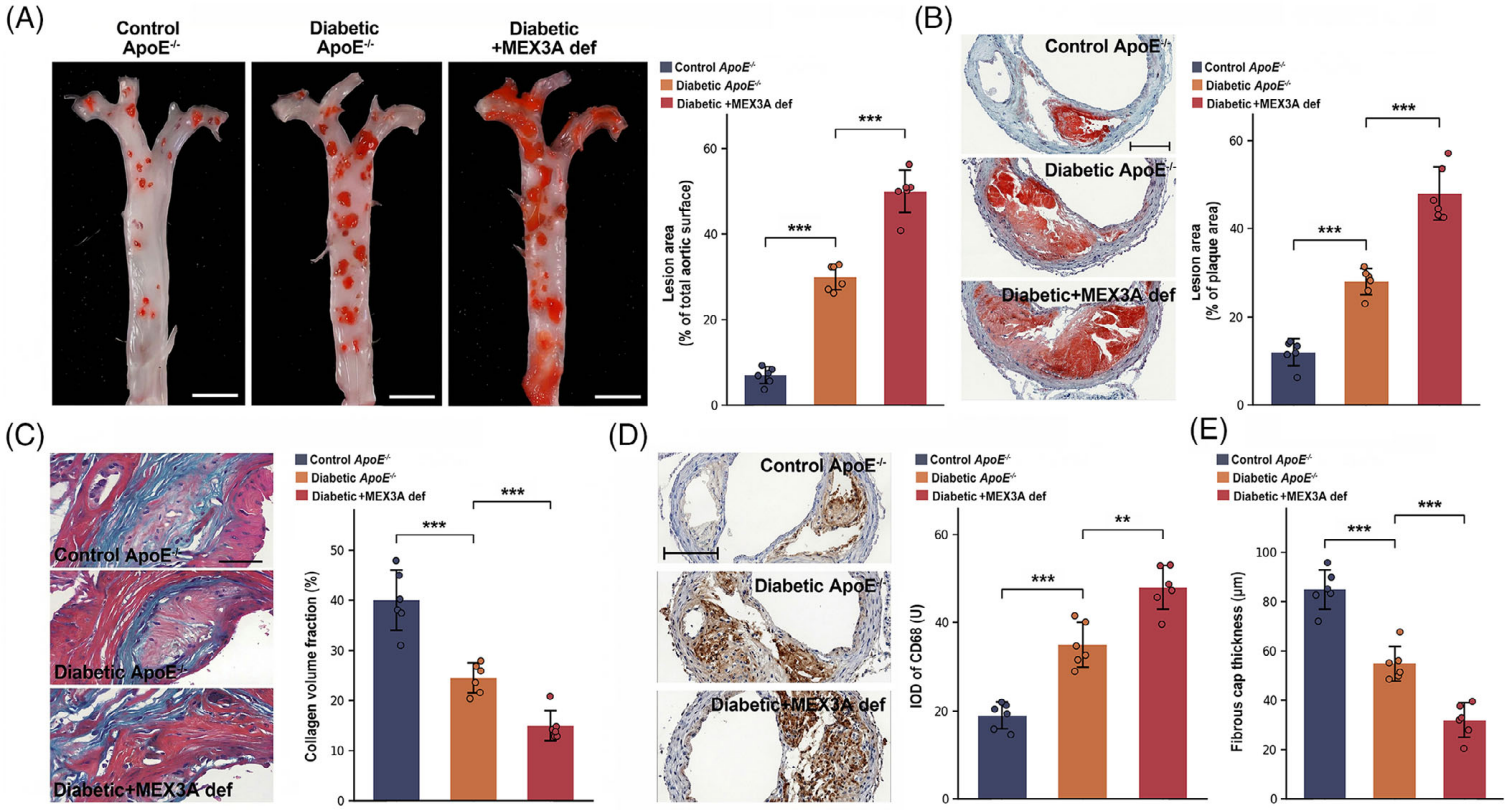
Mex‐3 RNA‐binding family member A (MEX3A) deficiency exacerbates plaque burden and promotes morphological features of plaque instability in diabetic atherosclerosis. (A) En face Oil Red O staining of whole aortas and quantification of lesion area as percentage of total aortic surface. Scale bars, 5 mm. (B) Oil Red O staining of aortic root cross‐sections and quantification of lesion area as percentage of plaque area. Scale bars, 100 µm. (C) Masson's trichrome staining and quantification of collagen volume fraction. Scale bars, 200 µm. (D) CD68 immunohistochemistry and quantification of CD68 immunoreactive signal. Scale bars, 100 µm. (E) Quantification of fibrous cap thickness. *n* = 6 mice per group. Data are mean ± standard deviation (SD). One‐way analysis of variance (ANOVA) with Tukey's post hoc test. ^**^ *p* < .01; ^***^ *p* < .001.

En face Oil Red O staining indicated that diabetes markedly promoted the aortic surface occupied by lesions compared with non‐diabetic *ApoE^−/−^* mice and that MEX3A deficiency further expanded lesion burden in the diabetic setting (Figure 2A). Aortic root Oil Red O staining suggested the same pattern: lipid‐rich plaque area increased in diabetic lesions and rose further after MEX3A loss (Figure 2B). Plaque composition changed in step with lesion enlargement. Masson's trichrome staining showed reduced collagen volume fraction in diabetic plaques, with an additional decline in MEX3A‐deficient diabetic lesions (Figure 2C). CD68 immunohistochemistry demonstrated higher macrophage accumulation in diabetic lesions and a further increment in MEX3A‐deficient plaques (Figure 2D). We also quantified established morphological features of plaque vulnerability. Fibrous cap thickness was reduced by diabetes and further reduced by MEX3A deficiency (Figure 2E), and supplementary morphometry showed that necrotic core area increased in parallel with lesion severity (Figure S1F,G). Thus, MEX3A restrains both the overall size of diabetic lesions and the compositional remodelling that favours unstable plaque morphology.

### 2.3 MEX3A deficiency amplifies ferroptosis‐associated injury in plaque macrophages

**FIGURE 3:**
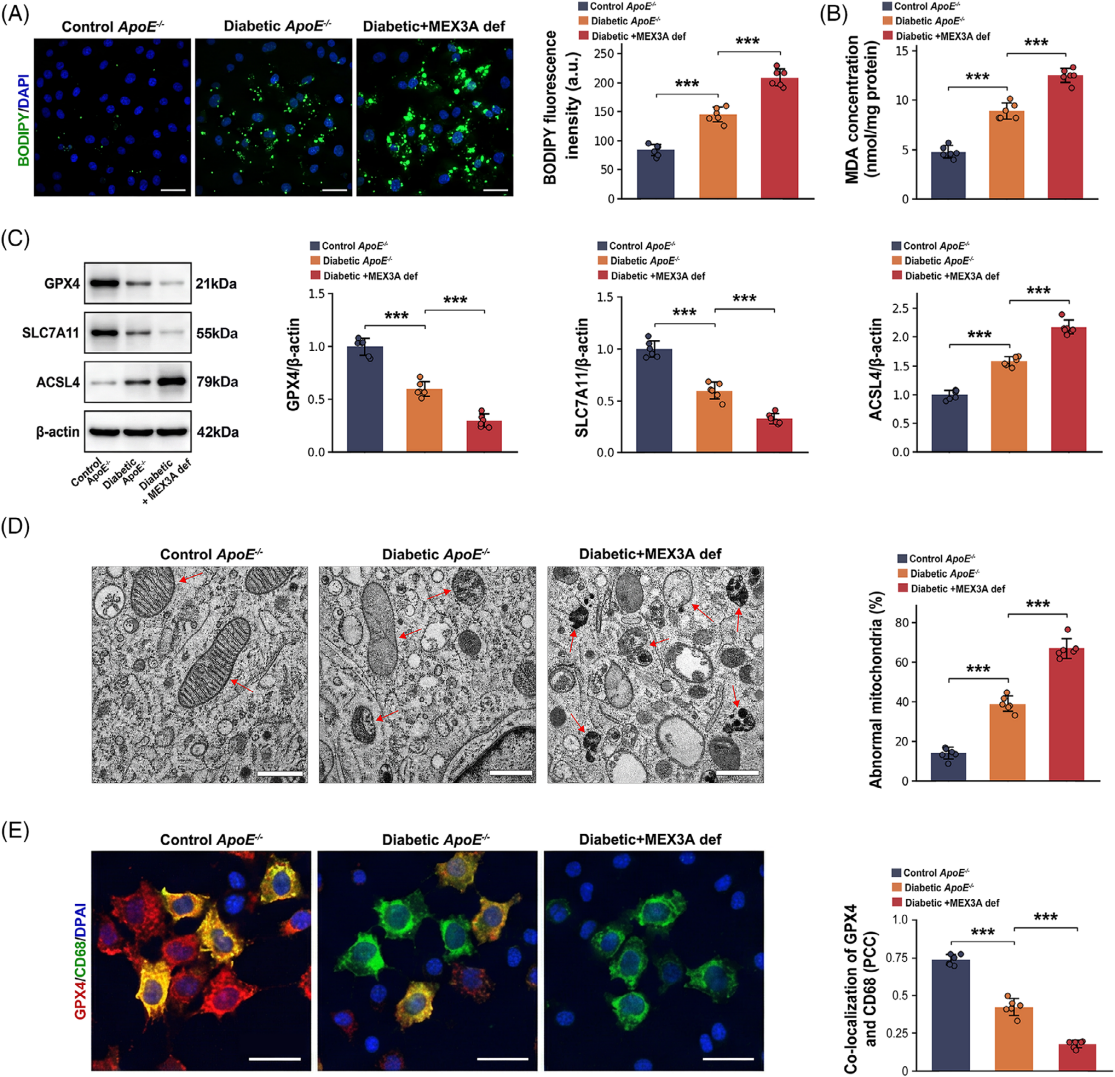
Mex‐3 RNA‐binding family member A (MEX3A) deficiency enhances ferroptosis‐associated injury in macrophages within diabetic atherosclerotic plaques. (A) BODIPY‐C11/4′,6‐diamidino‐2‐phenylindole (DAPI) fluorescence images and quantification of BODIPY‐C11 fluorescence intensity in aortic root plaques. Scale bars, 50 µm. (B) Aortic malondialdehyde (MDA) concentration. (C) Western blot analysis and quantification of GPX4, SLC7A11 and ACSL4 normalised to beta‐actin. (D) Transmission electron microscopy (TEM) images showing mitochondrial morphology and quantification of abnormal mitochondria. Red arrows indicate abnormal mitochondria. Scale bars, 500 nm. (E) Immunofluorescence images showing GPX4 (red), CD68 (green) and DAPI (blue) and quantification of GPX4/CD68 co‐localisation by Pearson correlation coefficient (PCC). Scale bars, 20 µm. *n* = 6 mice per group. Data are mean ± standard deviation (SD). One‐way analysis of variance (ANOVA) with Tukey's post hoc test. ^***^ *p* < .001.

Because plaque vulnerability was accompanied by increased macrophage burden, we next evaluated ferroptosis‐associated macrophage injury. BODIPY‐C11 imaging demonstrated higher lipid reactive oxygen species (ROS) in diabetic plaque macrophages than in controls and a further increase in diabetic MEX3A‐deficient lesions (Figure 3A). Aortic malondialdehyde (MDA) content was significantly elevated by diabetes and increased further with MEX3A deficiency (Figure 3B), indicating intensified tissue lipid peroxidation. Immunoblotting of aortic tissue identified a ferroptosis‐associated protein signature in MEX3A‐deficient diabetic mice: GPX4 and SLC7A11 were reduced, whereas ACSL4 was increased, relative to diabetic *ApoE* ^−/−^ controls (Figure 3C). Transmission electron microscopy (TEM) revealed contracted mitochondria, denser membranes and disrupted cristae in diabetic lesions, with more severe abnormalities in the setting of MEX3A deficiency (Figure 3D). In parallel, GPX4 immunofluorescence within CD68‐positive macrophages was decreased in diabetic plaques and fell further in MEX3A‐deficient diabetic lesions (Figure 3E). Together, these complementary readouts indicate that MEX3A deficiency intensifies ferroptosis‐associated stress in plaque macrophages.

### 2.4 CRISPR/Cas9‐mediated *Mex3a* knockout and MEX3A re‐expression define a *Mex3a*‐dependent macrophage phenotype

**FIGURE 4:**
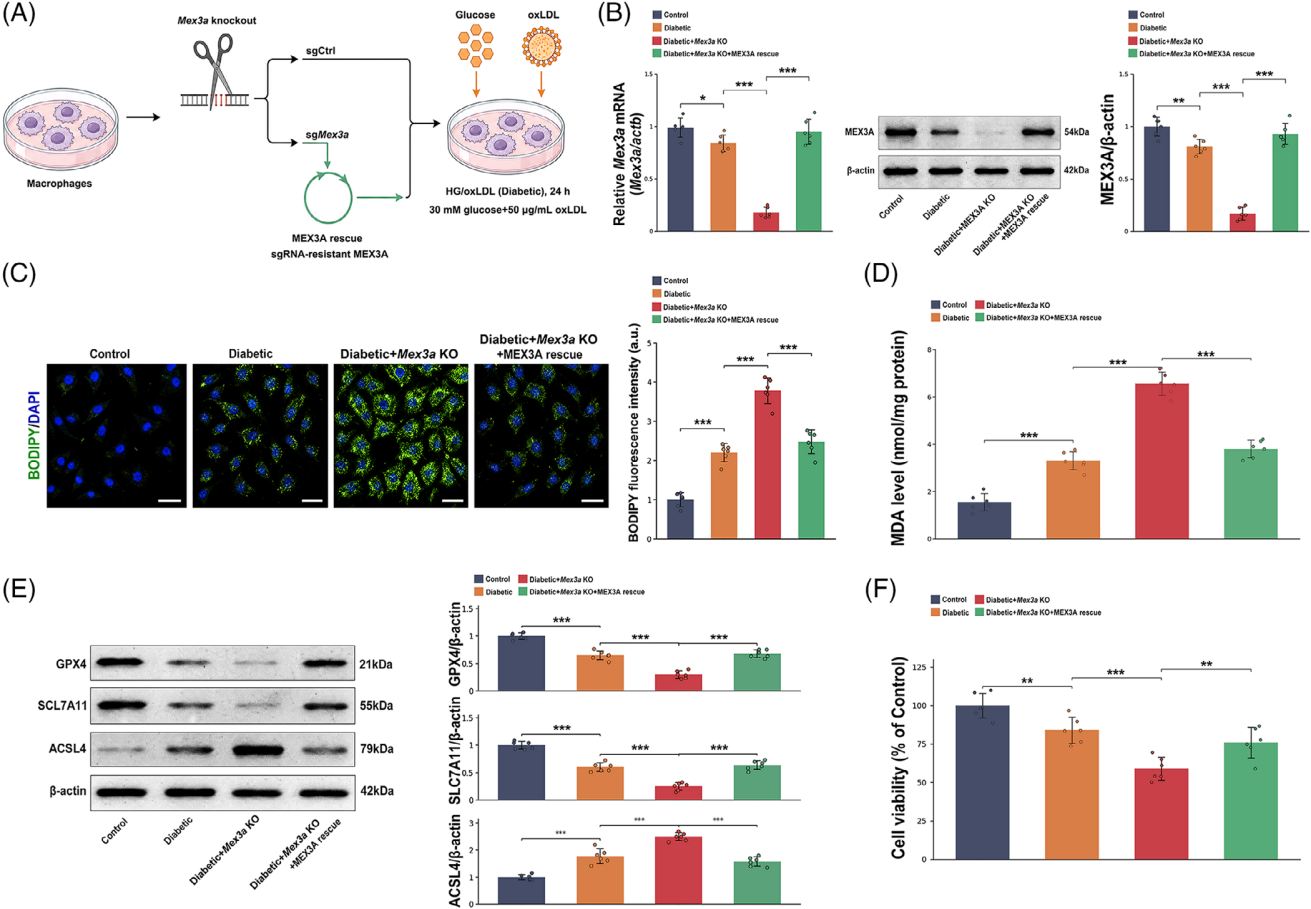
CRISPR/Cas9‐mediated *Mex3a* knockout and Mex‐3 RNA‐binding family member A (MEX3A) re‐expression define a *Mex3a*‐dependent macrophage phenotype. (A) Schematic of CRISPR/Cas9‐mediated *Mex3a* knockout, sgCtrl control, sg*Mex3a* targeting and sgRNA‐resistant MEX3A rescue in primary bone marrow‐derived macrophages (BMDMs) under high glucose/oxidised low‐density lipoprotein (HG/oxLDL) stimulation. (B) qPCR and Western blot verification of *Mex3a* knockout and MEX3A rescue. (C) BODIPY‐C11/4′,6‐diamidino‐2‐phenylindole (DAPI) fluorescence images and quantification of lipid reactive oxygen species (ROS). Scale bars, 50 µm. (D) Malondialdehyde (MDA) levels in BMDMs. (E) Western blot analysis and quantification of GPX4, SLC7A11 and ACSL4. (F) Cell Counting Kit‐8 (CCK‐8) cell viability assay. *n* = 6 independent BMDM preparations per group. Data are mean ± standard deviation (SD). One‐way analysis of variance (ANOVA) with Tukey's post hoc test. ^*^ *p* < .05; ^**^ *p* < .01; ^***^ *p* < .001.

To determine whether MEX3A itself controls this macrophage phenotype, we established CRISPR/Cas9‐mediated *Mex3a* knockout in primary BMDMs and then re‐expressed sgRNA‐resistant MEX3A before high glucose/oxidised low‐density lipoprotein (HG/oxLDL) stimulation (Figure 4A). qPCR and immunoblotting verified effective *Mex3a* depletion and restoration of MEX3A expression after rescue (Figure 4B). Under HG/oxLDL stimulation, *Mex3a* knockout markedly increased BODIPY‐C11 lipid ROS and MDA accumulation, whereas MEX3A re‐expression significantly attenuated these changes (Figure 4C,D). Consistently, *Mex3a* knockout suppressed GPX4 and SLC7A11 and increased ACSL4, while MEX3A rescue partially normalised these ferroptosis‐associated proteins (Figure 4E). Cell Counting Kit‐8 (CCK‐8) viability measurement further indicated that *Mex3a* knockout aggravated HG/oxLDL‐associated viability loss and that MEX3A re‐expression partially restored cell viability (Figure 4F). The HG/oxLDL condition itself caused only a moderate viability reduction in control macrophages and robustly induced BODIPY‐C11 oxidation without causing extensive non‐specific cytotoxicity (Figure S1A,B). Additional ferroptosis‐related endpoints, including labile Fe^2+^, reduced glutathione/oxidised glutathione (GSH/GSSG) ratio and 4‐hydroxynonenal (4‐HNE), also supported the same direction of effect and rescue (Figure S1C‒E).

### 2.5 MEX3A associates with *Slc7a11* and *Gpx4* transcripts and GPX4/SLC7A11 function downstream of the MEX3A‐linked phenotype

**FIGURE 5:**
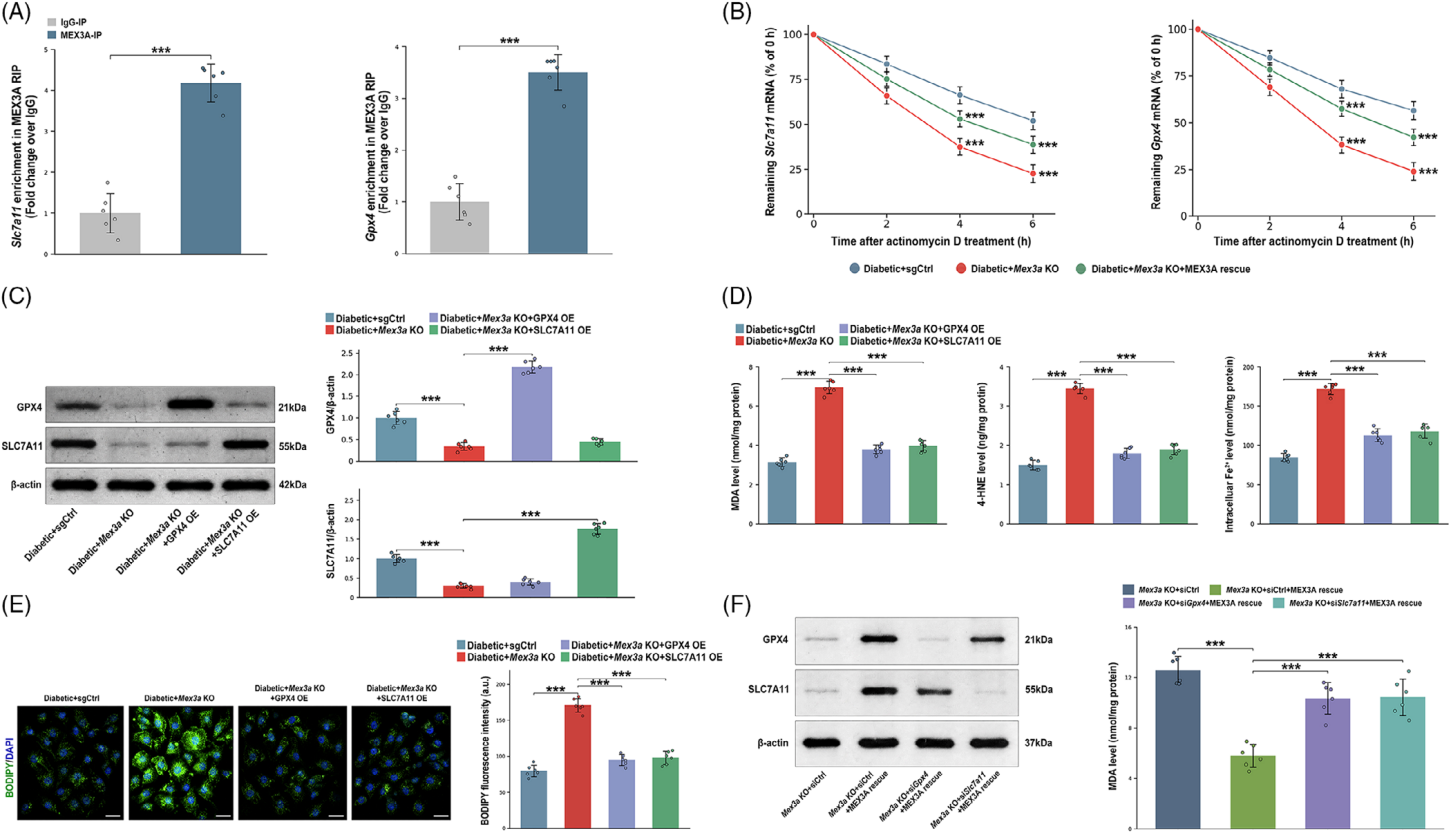
Mex‐3 RNA‐binding family member A (MEX3A) associates with *Slc7a11*/Gpx4 transcripts and GPX4/SLC7A11 mediate the MEX3A‐linked macrophage phenotype. (A) RNA immunoprecipitation‐qPCR (RIP‐qPCR) analysis showing *Slc7a11* and Gpx4 RNA enrichment in MEX3A immunoprecipitates relative to IgG control. (B) Actinomycin D chase assays showing remaining *Slc7a11* and Gpx4 mRNA percentages after transcriptional blockade in diabetic + sgCtrl, diabetic + *Mex3a*‐knockout (KO) and diabetic + *Mex3a*‐KO + MEX3A rescue macrophages. (C) Western blot validation of GPX4 or SLC7A11 overexpression in *Mex3a*‐KO bone marrow‐derived macrophages (BMDMs). (D) Malondialdehyde (MDA), 4‐hydroxynonenal (4‐HNE) and intracellular Fe^2+^ measurements after GPX4 or SLC7A11 overexpression. (E) BODIPY‐C11/4′,6‐diamidino‐2‐phenylindole (DAPI) images and quantification of lipid reactive oxygen species (ROS) after GPX4 or SLC7A11 overexpression. Scale bars, 50 µm. (F) Western blot verification of Gpx4 or *Slc7a11* knockdown in MEX3A‐rescued *Mex3a*‐KO cells and corresponding MDA readout. *n* = 6 independent BMDM preparations per group. Data are mean ± standard deviation (SD). One‐way or two‐way analysis of variance (ANOVA) with Tukey's post hoc test as appropriate. ^**^ *p* < .01; ^***^ *p* < .001.

Because MEX3A binds RNA, we next tested whether it engages ferroptosis‐related transcripts post‐transcriptionally. RNA immunoprecipitation‐qPCR (RIP‐qPCR) detected greater recovery of *Slc7a11* and *Gpx4* mRNAs with MEX3A than with immunoglobulin G (IgG) control pulldown (Figure 5A). Actinomycin D time‐course assays further showed faster loss of *Slc7a11* and *Gpx4* mRNAs after *Mex3a* knockout, whereas MEX3A re‐expression partially restored transcript stability (Figure 5B). The role of MEX3A in post‐transcriptional preservation of the SLC7A11/GPX4 antioxidant defence programme is supported by the findings. To test downstream sufficiency, GPX4 or SLC7A11 was overexpressed in *Mex3a*‐knockout macrophages. Western blotting confirmed successful overexpression (Figure 5C), and both GPX4 and SLC7A11 overexpression reduced MDA, 4‐HNE, intracellular Fe^2+^ and BODIPY‐C11 lipid ROS induced by *Mex3a* knockout (Figure 5D,E). To test downstream necessity for the rescue phenotype, *Gpx4* or *Slc7a11* was knocked down in MEX3A‐rescued *Mex3a*‐knockout macrophages. Knockdown of either transcript increased MDA relative to MEX3A rescue with control small interfering RNA (siRNA), indicating that GPX4 and SLC7A11 contribute to the protection conferred by MEX3A re‐expression (Figure 5F).

### 2.6 Ferrostatin‐1 attenuates MEX3A deficiency‐associated ferroptosis‐related injury in macrophages under diabetic conditions

**FIGURE 6:**
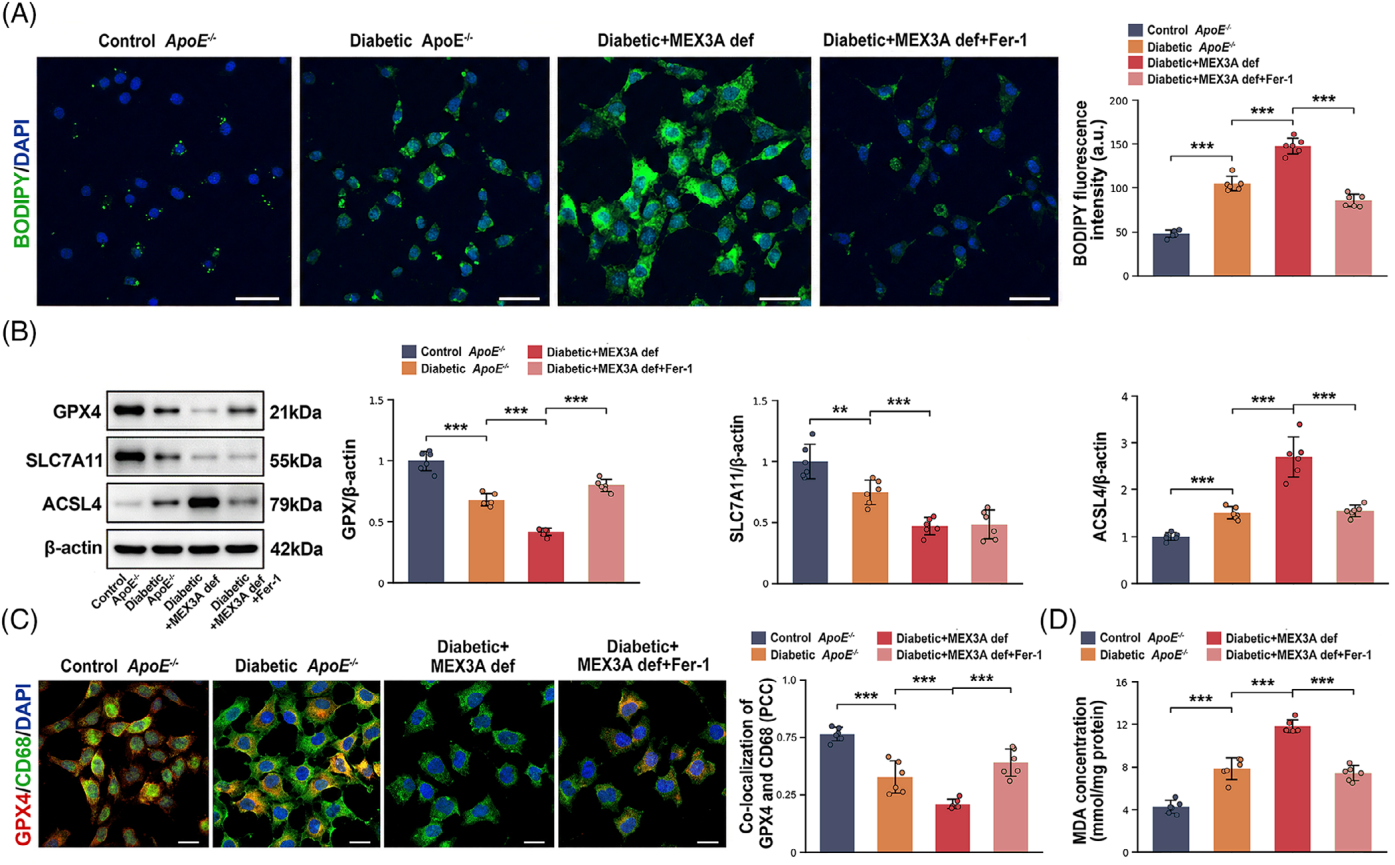
Ferrostatin‐1 (Fer‐1) attenuates Mex‐3 RNA‐binding family member A (MEX3A) deficiency‐associated ferroptosis‐related injury in macrophages under diabetic conditions. (A) BODIPY‐C11/4′,6‐diamidino‐2‐phenylindole (DAPI) fluorescence images and quantification of BODIPY‐C11 fluorescence intensity in control *ApoE* ^−/−^, diabetic *ApoE* ^−/−^, diabetic + MEX3A def and diabetic + MEX3A def + Fer‐1 macrophages. Scale bars, 50 µm. (B) Western blot analysis and quantification of GPX4, SLC7A11 and ACSL4 normalised to beta‐actin. (C) Immunofluorescence images showing GPX4 (red), CD68 (green) and DAPI (blue), with quantification of GPX4/CD68 co‐localisation by Pearson correlation coefficient (PCC). Scale bars, 20 µm. (D) Malondialdehyde (MDA) concentration. *n* = 6 independent bone marrow‐derived macrophage (BMDM) preparations per group. Data are mean ± standard deviation (SD). One‐way analysis of variance (ANOVA) with Tukey's post hoc test. ^**^ *p* < .01; ^***^ *p* < .001.

We next applied Fer‐1 to test whether blocking ferroptosis pharmacologically could reduce the macrophage injury phenotype. In vitro BODIPY‐C11 imaging showed that diabetic stress increased lipid ROS, MEX3A deficiency amplified this response and Fer‐1 suppressed the excess lipid peroxidation associated with MEX3A loss (Figure 6A). Immunoblotting demonstrated that MEX3A deficiency under diabetic conditions decreased GPX4 and SLC7A11 while increasing ACSL4, reproducing the ferroptosis‐associated pattern observed in vivo. Fer‐1 partially normalised GPX4‐related signals and reduced ACSL4 abundance (Figure 6B). GPX4/CD68 co‐localisation was reduced by diabetic stress and further diminished by MEX3A deficiency, whereas Fer‐1 partially increased macrophage‐associated GPX4 signal (Figure 6C). Fer‐1 also lowered intracellular MDA accumulation (Figure 6D). These data indicate that ferroptosis‐associated lipid peroxidation contributes to the oxidative phenotype caused by MEX3A deficiency; however, Fer‐1 acts downstream of MEX3A and attenuates injury rather than restoring MEX3A itself.

### 2.7 Ferrostatin‐1 partially attenuates plaque destabilisation associated with MEX3A deficiency in vivo

**FIGURE 7:**
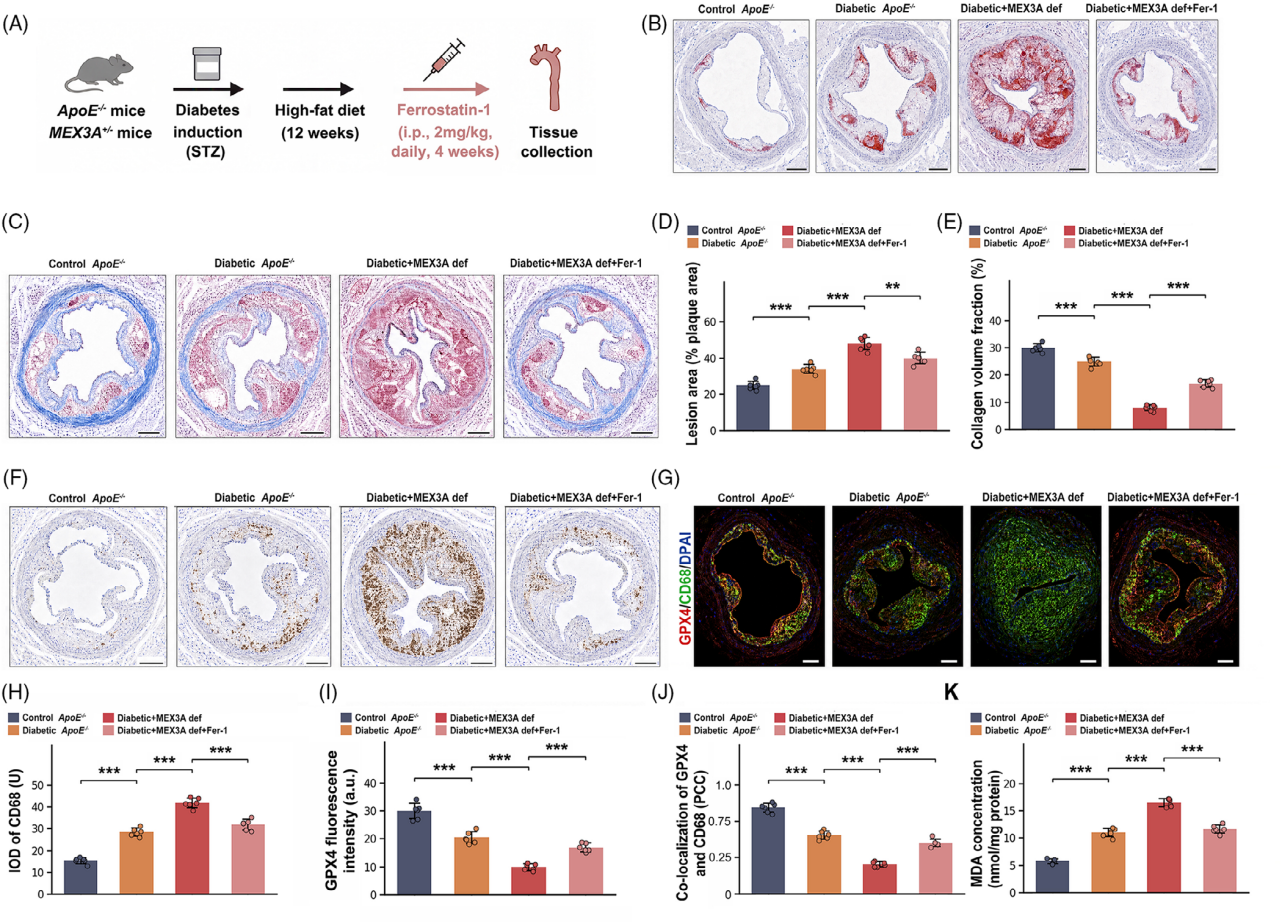
Ferrostatin‐1 (Fer‐1) partially attenuates plaque destabilisation associated with Mex‐3 RNA‐binding family member A (MEX3A) deficiency in vivo. (A) Schematic of the in vivo Fer‐1 treatment design. Diabetic *ApoE* ^−/−^ *Mex3a* ^+/−^ mice were treated with Fer‐1 (2 mg/kg, intraperitoneal injection, daily) for the final 4 weeks of high‐fat diet (HFD) feeding. (B and D) Oil Red O staining of aortic root sections and lesion area quantification. Scale bars, 200 µm. (C and E) Masson's trichrome staining and collagen volume fraction quantification. Scale bars, 200 µm. (F and H) CD68 immunohistochemistry and quantification. Scale bars, 200 µm. (G, I, and J) Immunofluorescence images showing GPX4 (red), CD68 (green) and 4′,6‐diamidino‐2‐phenylindole (DAPI) (blue), with quantification of GPX4 fluorescence intensity and GPX4/CD68 co‐localisation by Pearson correlation coefficient (PCC). Scale bars, 200 µm. (K) Aortic malondialdehyde (MDA) concentration. *n* = 6 mice per group. Data are mean ± standard deviation (SD). One‐way analysis of variance (ANOVA) with Tukey's post hoc test. ^**^ *p* < .01; ^***^ *p* < .001.

Finally, we tested whether ferroptosis inhibition could lessen the vascular consequences of MEX3A deficiency during diabetic atherosclerosis in vivo. Diabetic *ApoE* ^−/−^ *Mex3a* ^+/−^ mice were treated with Fer‐1 during the late phase of lesion development (Figure 7A). Aortic root Oil Red O staining demonstrated reduced lipid‐rich plaque area after Fer‐1 treatment relative to untreated diabetic MEX3A‐deficient mice (Figure 7B,D). Plaque composition also shifted towards a more stable phenotype. Fer‐1 partially restored collagen content within lesions (Figure 7C,E) and reduced CD68‐positive macrophage accumulation (Figure 7F,H). GPX4 fluorescence intensity and GPX4/CD68 co‐localisation were increased after Fer‐1 treatment (Figure 7G,I,J), indicating partial recovery of macrophage antioxidant capacity in situ. At the biochemical level, Fer‐1 lowered aortic MDA content (Figure 7K), consistent with suppression of lipid peroxidation. The attenuation was partial rather than complete, suggesting that MEX3A deficiency likely influences diabetic plaque biology through ferroptosis‐associated and ferroptosis‐independent mechanisms. Nevertheless, these data establish ferroptosis‐associated lipid peroxidation as a therapeutically actionable effector process in MEX3A‐deficient diabetic atherosclerosis.

## 3 DISCUSSION

This study positions MEX3A as a protective factor in diabetic plaque biology and connects its loss with ferroptosis‐associated macrophage injury.^13^, ^17^ Across genetic, biochemical, ultrastructural and pharmacological analyses, MEX3A deficiency consistently intensified diabetic lesion burden, increased macrophage content, reduced collagen, expanded necrotic cores and thinned fibrous caps.^3^, ^4^ These pathological changes were accompanied by excess lipid peroxidation, lower GPX4 and SLC7A11, higher ACSL4 and mitochondrial injury.^6^, ^7^ Targeted macrophage experiments further showed that CRISPR/Cas9‐mediated *Mex3a* knockout reproduced the phenotype and that MEX3A re‐expression attenuated it, supporting a *Mex3a*‐dependent macrophage phenotype.

Mechanistically, the data link MEX3A to post‐transcriptional maintenance of the SLC7A11/GPX4 antioxidant programme in macrophages. MEX3A RIP‐qPCR recovered *Slc7a11* and *Gpx4* transcripts, and actinomycin D chase assays showed faster decay of these transcripts after *Mex3a* knockout with partial stabilisation after MEX3A re‐expression.^14^, ^15^ Functionally, GPX4 or SLC7A11 overexpression attenuated MDA accumulation, 4‐HNE formation, labile Fe^2+^ accumulation and BODIPY‐C11 oxidation in *Mex3a*‐knockout macrophages, whereas *Gpx4* or *Slc7a11* knockdown diminished the protection produced by MEX3A re‐expression.^10^, ^11^ These findings support an MEX3A‐associated post‐transcriptional mechanism that helps preserve the SLC7A11/GPX4 antioxidant defence programme. The data do not exclude additional RNA targets of MEX3A, and transcriptome‐wide binding studies will be important for defining the broader MEX3A‐regulated network in plaque macrophages.

Fer‐1 provided complementary functional evidence that lipid‐peroxidation‐driven ferroptotic injury contributes to the MEX3A‐deficient phenotype. Fer‐1 is a downstream ferroptosis inhibitor and did not restore MEX3A expression; accordingly, its protective effect represents attenuation of ferroptosis‐associated injury rather than correction of the primary genetic defect.^6^, ^8^ The incomplete protection observed in cultured macrophages and diabetic plaques indicates that MEX3A likely modulates additional macrophage processes beyond ferroptosis. Candidate processes include inflammatory activation, efferocytosis, lipid handling, inflammasome signalling, apoptosis and necroptosis, each of which can influence plaque progression and stability.^5^ Thus, the current data support ferroptosis‐associated lipid peroxidation as an important effector of MEX3A deficiency without establishing it as the sole mechanism of plaque destabilisation.^13^, ^17^

The model system and phenotype analyses define the biological context of these findings. The HG/oxLDL stimulus induced BODIPY‐C11 oxidation without causing extensive non‐specific cytotoxicity, supporting its use as a macrophage diabetic‐stress model. Labile Fe^2+^, glutathione/glutathione disulphide ratio and 4‐HNE provided further evidence of iron‐linked redox imbalance and lipid peroxidation.^6^, ^10^ Plaque stability was evaluated using accepted morphological indices, including fibrous cap thickness and necrotic core percentage, which complement lipid deposition, collagen content and macrophage abundance.^3^, ^4^ Consistent magnification and scale bars were used for ultrastructural comparisons, and all quantitative analyses were performed with predefined biological replicate numbers.

This work has limitations. The in vivo model used systemic MEX3A heterozygous deficiency on the *ApoE* ^−/−^ background rather than macrophage‐specific deletion, so contributions from endothelial cells, vascular smooth muscle cells, hepatometabolic tissues or other immune compartments cannot be excluded.^4^, ^16^ The animal model used young adult male mice and represents an accelerated experimental model of diabetic atherosclerosis; it cannot fully recapitulate the chronic age‐associated evolution of human atherosclerosis in middle‐aged or elderly patients with diabetes.^1^, ^2^ Although MEX3A binding to *Slc7a11* and *Gpx4* transcripts was supported by RIP‐qPCR, transcriptome‐wide CLIP‐sequencing and macrophage‐specific in vivo rescue models will be needed in future studies to define direct binding sites and cell‐type specificity.^14^, ^15^ Even with these caveats, the current data establish a disease‐relevant link between MEX3A loss, impaired SLC7A11/GPX4 antioxidant defence, macrophage ferroptosis‐associated injury and diabetic plaque instability.^13^, ^17^

## 4 MATERIALS AND METHODS

### 4.1 Study design, sample size, randomisation and blinding

The study integrated an in vivo diabetic atherosclerosis model, ex vivo tissue analyses and in vitro mechanistic experiments in primary BMDMs. The prespecified primary endpoints were aortic lesion burden, aortic root lipid deposition, collagen content, macrophage accumulation, fibrous cap thickness, necrotic core area, lipid peroxidation and ferroptosis‐related protein expression. Sample size was calculated from pilot aortic root lesion‐area and MDA data, using alpha = .05 and 80% power; a minimum of five biological replicates per group was required, and six mice or six independent cell preparations were used where feasible to account for attrition. A computer‐generated random sequence was used to allocate animals and cell preparations to experimental groups. Investigators performing tissue sectioning, histological scoring, immunofluorescence quantification, TEM analysis and biochemical quantification were blinded to group allocation until final data analysis. Data points were retained unless a prespecified technical failure occurred, such as tissue loss during sectioning, inadequate fixation or failed positive/negative assay controls.

### 4.2 Animals, sex and ethics

All animal work followed the Guide for the Care and Use of Laboratory Animals. The protocol was reviewed and approved by the Animal Experimental Ethics Committee of Northwestern Polytechnical University (approval no. 202602008). Male = ApoE^−/−^ mice on the C57BL/6J background (The Jackson Laboratory) were crossed with male or female *Mex3a* heterozygous knockout mice (*Mex3a* ^+/−^, CRISPR/Cas9‐generated, C57BL/6J background; Cyagen Biosciences) to generate ApoE^−/−^ *Mex3a* ^+/−^ offspring. Age‐matched male littermate ApoE^−/−^ mice were used as controls. Mice were maintained under SPF conditions at 22 ± 2°C with a 12 h artificial light/dark cycle, 50%–60% humidity and ad libitum access to water and food.

### 4.3 Genotyping

Tail‐biopsy genomic DNA was isolated with the DNeasy Blood & Tissue Kit (Qiagen). PCR amplification used TaKaRa Taq DNA Polymerase (Takara) on a T100 Thermal Cycler (Bio‐Rad). The primers were *Mex3a*‐F, 5′‐CCGGTCTCATCGTCAACTAC‐3′, and *Mex3a*‐R, 5′‐GCTTGCTGTAGGGTCTGTAG‐3′. PCR amplicons were resolved on 1.5% agarose gels containing GelRed nucleic acid stain (Biotium).The ChemiDoc MP Imaging System (Bio‐Rad) was used to acquire the image.

### 4.4 Induction of diabetic atherosclerosis

At 8 weeks of age, mice were made diabetic with STZ (Sigma‒Aldrich) freshly dissolved in .1 M cold citrate buffer (pH 4.5). STZ (50 mg/kg) was injected intraperitoneally daily for 5 consecutive days. Vehicle alone was given to control mice. After a 6 h fast, tail‐vein blood was used to measure glucose by OneTouch glucometer (LifeScan). Diabetes was defined as fasting glucose ≥16.7 mmol/L on two consecutive measurements. After diabetes was confirmed, mice were switched to an HFD (21% fat and .15% cholesterol, #D12108C, Research Diets Inc.) for the following 12 weeks. Plasma total cholesterol and triglycerides were quantified with LabAssay Cholesterol and LabAssay Triglyceride kits (FUJIFILM).

### 4.5 Ferrostatin‐1 treatment in vivo

For in vivo inhibition of ferroptosis, diabetic ApoE^−/−^ *Mex3a* ^+/−^ mice received Fer‐1 (Selleck Chemicals) at 2 mg/kg by intraperitoneal injection once daily for the final 4 weeks of HFD feeding. Fer‐1 was prepared in .5% carboxymethylcellulose sodium (Sigma‒Aldrich) containing 1% dimethyl sulphoxide (Sigma‒Aldrich) and administered in 100 µL volume. Vehicle‐treated animals were administrated with an equal volume on the same schedule.

### 4.6 Primary macrophage isolation, differentiation and diabetic stimulation

Primary BMDMs were obtained from femurs and tibiae of 8‐week‐old male mice. Bone marrow cells were differentiated for 7 days in Dulbecco's modified Eagle's medium (Gibco, Thermo Fisher Scientific) supplemented with 10% foetal bovine serum (HyClone, Cytiva), 1% penicillin‒streptomycin (Gibco) and 20 ng/mL recombinant mouse macrophage colony‐stimulating factor (PeproTech). Cells were cultured at 37°C in humidified 5% CO_2_ (Thermo Fisher Scientific). Diabetic‐like conditions were modelled by exposing cells to 30 mM D‐glucose (Sigma‒Aldrich) plus 50 µg/mL oxLDL (Shenggong) for 24 h. Osmotic control cells were treated with 5.5 mmol/L glucose plus 24.5 mmol/L mannitol (Sigma‒Aldrich). When specified, cells received pretreatment with 1 µM Fer‐1 for 1 h before HG/oxLDL stimulation.

### 4.7 CRISPR/Cas9‐mediated *Mex3a* knockout and MEX3A rescue

For target‐specific validation, primary BMDMs received lentiviral CRISPR/Cas9 vectors encoding a non‐targeting single‐guide RNA (sgCtrl) or a *Mex3a*‐targeting single‐guide RNA (sg*Mex3a*). Lentiviral particles and the sgRNA‐resistant MEX3A rescue construct were generated by GeneChem. The sg*Mex3a* target sequence was 5′‐GCTGCTGATGACCTACGAGA‐3′; the non‐targeting control sequence was 5′‐GCGAGGTATTCGGCTCCGCG‐3′. Transduced cells underwent selection with 2 µg/mL puromycin (InvivoGen) for 72 h. For rescue experiments, *Mex3a*‐knockout BMDMs received an sgRNA‐resistant mouse MEX3A lentiviral expression vector (GeneChem) 48 h before HG/oxLDL stimulation. *Mex3a* knockout and MEX3A re‐expression were verified by qPCR and Western blotting.

### 4.8 GPX4/SLC7A11 overexpression and siRNA knockdown

For testing downstream mediators, *Mex3a*‐knockout BMDMs were transduced with lentiviral mouse GPX4 or SLC7A11 overexpression vectors (GeneChem); empty vector served as control. For loss‐of‐rescue experiments, MEX3A‐rescued *Mex3a*‐knockout BMDMs were transfected with siRNAs targeting *Gpx4* and *Slc7a11* or non‐targeting siCtrl (GenePharma) using Lipofectamine 3000 (Invitrogen) according to the manufacturer's protocol. The siRNA sequences were: si*Gpx4*, 5′‐GCAAGACCGAAGTAAACTA‐3′; si*Slc7a11*, 5′‐GGAGGTTACCTGCAGTTTA‐3′; siCtrl, 5′‐UUCUCCGAACGUGUCACGUTT‐3′. Overexpression and knockdown efficiency were verified by Western blotting before lipid‐peroxidation assays.

### 4.9 Cell viability assay

Cell viabilities were determined using CCK‐8 (Dojindo Laboratories). Treated BMDMs were incubated with CCK‐8 reagent at 37°C for 2 h. Then, the absorbance at 450 nm was measured by a SpectraMax iD3 microplate reader (Molecular Devices).

### 4.10 RNA extraction, reverse transcription and qPCR

Total RNA was extracted using TRIzol Reagent (Invitrogen, Thermo Fisher Scientific). Quantity and purity of RNA were evaluated using a NanoDrop 2000 spectrophotometer (Thermo Fisher Scientific). PrimeScript RT Reagent Kit (Takara Bio) was used to prepare complementary DNA (cDNA). qPCR reactions was performed by using the TB Green Premix Ex Taq II (Takara Bio) on a CFX96 Real‐Time PCR Detection System (Bio‐Rad). Relative mRNA abundance was calculated by the 2^△△CT^ method with Actb as the internal control. Primer sequences were: *Mex3a*‐F 5′‐AGGAGGACCTGCTGAAGAAA‐3′, *Mex3a*‐R 5′‐TCTTCTCGTAGGTCATCAGCAG‐3′; *Slc7a11*‐F 5′‐ATGCTGGTGGTGTATGCTTG‐3′, *Slc7a11*‐R 5′‐GCAAGAGGAGTGTGCTTGCG‐3′; *Gpx4*‐F 5′‐GCAACCAGTTTGGGAGGCAG‐3′, *Gpx4*‐R 5′‐CCTCCATGGGACCATAGCG‐3′; Actb‐F 5′‐GGCTGTATTCCCCTCCATCG‐3′, Actb‐R 5′‐CCAGTTGGTAACAATGCCATGT‐3′.

### 4.11 RNA immunoprecipitation‐qPCR

The Magna RIP RNA‐Binding Protein Immunoprecipitation Kit (Millipore) was used to perform RIP assays. BMDM lysates were combined with anti‐MEX3A antibody (Abcam) or normal rabbit IgG (Cell Signaling Technology) coupled to magnetic beads at 4°C for 10 h. Co‐precipitated RNA was purified according to the kit protocol and analysed by qPCR for *Slc7a11* and *Gpx4*. Readings were normalised to IgG immunoprecipitation and are reported as fold changes over IgG enrichment.

### 4.12 Actinomycin D mRNA stability assay

For mRNA decay measurements, BMDMs were exposed to 5 µg/mL actinomycin D (Sigma‒Aldrich) after HG/oxLDL stimulation. Cells were harvested 0, 2, 4 and 6 h after actinomycin D was added. *Slc7a11* and *Gpx4* mRNA levels were examined by qPCR.

### 4.13 Histology, plaque morphometry and image quantification

For en face analysis, harvested aortas were fixed with 4% paraformaldehyde (Shenggong) for 24 h and then stained with an Oil Red O staining kit (Sigma‒Aldrich). Images were obtained by Pannoramic 250 Flash III slide scanner (3DHISTECH). Lesion burden was quantified as the Oil Red O‐positive stained area of the total aortic surface. For aortic root analysis, harvested hearts were fixed in optimal cutting temperature compound (Sakura Finetek) and cut into serial 8 µm cryo sections by Leica CM1950 cryostat (Leica Biosystems). Aortic root sections underwent Oil Red O, haematoxylin and eosin (Servicebio) or Masson's trichrome staining kits (Abcam). Collagen volume fraction was defined as collagen‐positive area divided by plaque area. Fibrous cap thickness was measured at the thinnest cap region from the luminal surface to the necrotic core boundary. Necrotic core area was defined as acellular or hypocellular plaque area and expressed as the percentage of total plaque area. Two blinded investigators averaged measurements from three serial sections per mouse.

### 4.14 Immunohistochemistry and immunofluorescence

For immunohistochemistry, tissue sections were subjected to heat‐induced antigen retrieval in 10 mmol/L citrate buffer (pH 6.0; Servicebio), blocked in 5% bovine serum albumin (BSA, Sigma‒Aldrich), and then incubated with anti‐CD68 antibody (Abcam, ab125212, 1:200) for 10 h at 4°C. Signal was visualised with a DAB kit (Vector Laboratories). For immunofluorescence, tissue sections or harvested BMDMs were fixed with 4% paraformaldehyde, permeabilised with .1% Triton X‐100 (Sigma‒Aldrich), blocked with 5% BSA. Primary antibodies against MEX3A (Abcam, ab155240, 1:100), GPX4 (Abcam, ab125066, 1:200), CD68 (Abcam, ab125212, 1:200) or 4‐HNE (Abcam, ab46545, 1:200) were used to incubate the sections. Alexa Fluor 488‐, 555‐ or 647‐conjugated secondary antibodies (Invitrogen, Thermo Fisher Scientific) were used. Nuclei were counterstained with 4′,6‐diamidino‐2‐phenylindole (Sigma‒Aldrich). Zeiss LSM 800 confocal microscope (Carl Zeiss) was used to observe and acquire images. Fluorescence intensity and Pearson correlation coefficient were quantified using ImageJ software.

### 4.15 Lipid ROS, labile Fe^2+^, GSH/GSSG, 4‐HNE and MDA assays

Lipid ROS was assessed with BODIPY 581/591 C11 (Thermo Fisher Scientific). Cells were loaded with 5 µmmol/L dye for 30 min at 37°C, washed with phosphate‐buffered saline (Gibco) and imaged by confocal microscopy. Labile Fe^2+^ was visualised with FerroOrange (Dojindo Laboratories) according to the protocol provided by the manufacturer. The GSH/GSSG ratio was measured using the GSH/GSSG Assay Kit (Beyotime Biotechnology). 4‐HNE was detected by immunofluorescence using anti‐4‐HNE antibody as described above. Tissue and cellular MDA content was quantified with a MDA assay kit (Cayman Chemical) and normalised to total protein concentration which was determined by the Pierce BCA Protein Assay Kit (Thermo Fisher Scientific).

### 4.16 Western blotting

Samples were homogenised in radioimmunoprecipitation assay buffer system (Beyotime Biotechnology) containing protease and phosphatase inhibitor cocktail (Roche). Protein concentration was determined with the Pierce BCA Protein Assay Kit. Equal protein amounts (30 µg per lane) were resolved by sodium dodecyl sulphate‒polyacrylamide gel electrophoresis and transferred to polyvinylidene fluoride membranes (Millipore). Membranes were blocked in 5% non‐fat milk (BD Difco) and incubated with anti‐MEX3A (Abcam, ab155240, 1:1000), anti‐GPX4 (Abcam, ab125066, 1:1000), anti‐SLC7A11 (Cell Signaling Technology, 12691, 1:1000), anti‐ACSL4 (Abcam, ab155282, 1:1000) and anti‐beta‐actin (Sigma‒Aldrich, A5441, 1:5000). Horseradish peroxidase (HRP)‐conjugated anti‐rabbit IgG (Cell Signaling Technology, 7074, 1:2000) and anti‐mouse IgG (Cell Signaling Technology, 7076, 1:2000) were used as secondary antibodies. Blots were visualised using Immobilon Western Chemiluminescent HRP Substrate (Millipore) and captured using a ChemiDoc MP Imaging System.

### 4.17 Transmission electron microscopy

Aortic root plaque samples were fixed with 2.5% glutaraldehyde (Solarbio) in .1 M phosphate buffer, post‐fixed in 1% osmium tetroxide (Solarbio), passed through graded ethanol for dehydration and then embedded in epoxy resin (Servicebio). Ultrathin sections (70 nm) were placed on copper grids and examined with a JEM‐1400 Plus transmission electron microscope (JEOL) at 80 kV. Comparable magnification and calibrated 500 nm scale bars were used across groups. Mitochondria were classified as abnormal when they exhibited shrinkage, increased membrane density or cristae disruption. Mitochondrial morphology was quantified in 10 random fields per sample by a blinded investigator.

### 4.18 Statistical analysis

Data are presented as mean ± standard deviation. Statistical analyses were performed using GraphPad Prism 9.0 (GraphPad Software). Comparisons among multiple groups were performed using one‐way analysis of variance (ANOVA) followed by Tukey's post hoc test. Time‐course data from actinomycin D chase assays were analysed using two‐way ANOVA followed by Tukey's post hoc test. A *p* < .05 was considered statistically significant.

## AUTHOR CONTRIBUTIONS

*Conceptualisation, methodology, investigation, formal analysis, data curation, visualisation and writing—original draft*: Yanpeng Ma. *Investigation, methodology and data curation*: Jing Liu. *Investigation and formal analysis*: Yujie Xing. *Investigation and resources*: Shuo Pan. *Formal analysis and validation*: Begench H. Annayev. *Resources and supervision*: We Wu. *Supervision, funding acquisition and project administration*: Xiqiang Wang. *Conceptualisation, supervision, funding acquisition and writing—review and editing*: Zhongwei Liu.

## CONFLICT OF INTEREST STATEMENT

The authors declare they have no conflicts of interest.

## ETHICS STATEMENT

Animal Experimental Ethics Committee of Northwestern Polytechnical University reviewed and approved all animal experiments protocols (no. 202602008).

## Supporting information



Supporting Information

## Data Availability

Data supporting this study are available from the corresponding author on reasonable request. No public transcriptomic, proteomic or imaging dataset was analysed in this study; therefore, no public data accession number or repository link is applicable. This study did not generate custom code. Standard ImageJ/Fiji and GraphPad Prism workflows were used for image and statistical analyses.
