# Erratum to “Mex‐3 RNA‐binding family member A limits macrophage ferroptosis‐associated injury linked to the SLC7A11/GPX4 pathway in diabetic atherosclerosis”

**DOI:** 10.1002/ctm2.70747

**Published:** 2026-07-14

Ma Y, Liu J, Xing Y, Mex‐3 RNA‐binding family member A limits macrophage ferroptosis‐associated injury linked to the SLC7A11/GPX4 pathway in diabetic atherosclerosis. *Clin. Transl. Med*. 2026;16:e70725. doi:10.1002/ctm2.70725.

In the originally published version of this article, the author name “We Wu” in the author list and Correspondence section was incorrect. This should have read: “Wei Wu”. This correction concerns only the spelling of the author name and does not affect the authorship order, author affiliations, author contributions, scientific content, data interpretation, or conclusions of the article.

We apologize for this error.

