# Supplementary figures and images for: Mex‐3 RNA‐binding family member A limits macrophage ferroptosis‐associated injury linked to the SLC7A11/GPX4 pathway in diabetic atherosclerosis
Source: Clin Transl Med. 2026 Jun 22;16(7):e70725. doi: 10.1002/ctm2.70725 (PMC13287327; doi:10.1002/ctm2.70725)

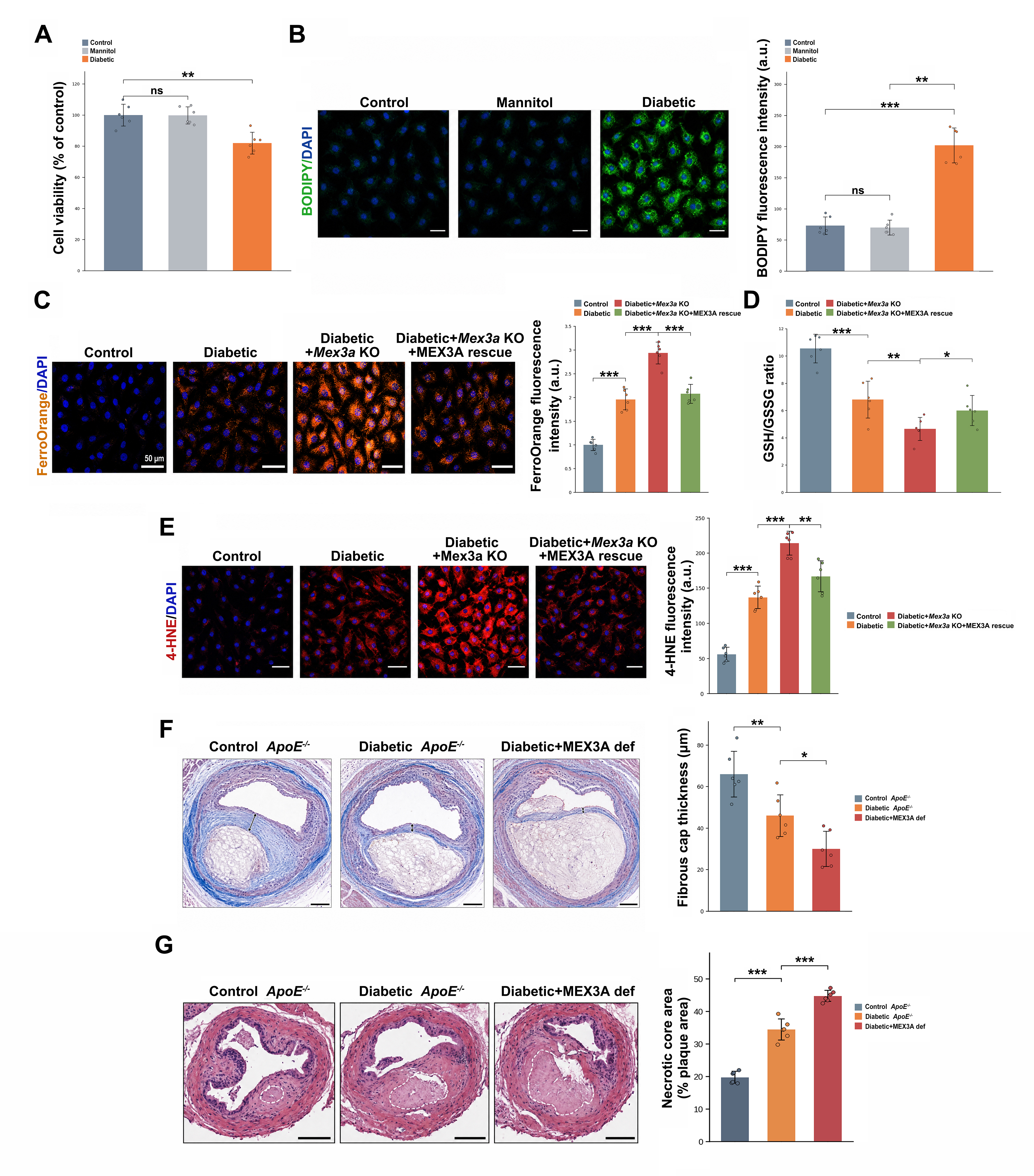

Supplement: Supplementary file 1 — Supporting Information [file CTM2-16-e70725-s001.jpg]
